# Supplementary material for: Comparative analysis of wavelet transform filtering systems for noise reduction in ultrasound images
Source: PLoS One. 2022 Jul 7;17(7):e0270745. doi: 10.1371/journal.pone.0270745 (PMC9262246; doi:10.1371/journal.pone.0270745)
Supplement: Attachment — Submitted filename: Response to Reviewers.pdf [file pone.0270745.s001.pdf]

Dear Editor,

Thank you for you feedback to our manuscript.

Below, we are uploading point-by-point response to the comments of all reviewers. We kept the changes after the first submit highlighted in red and the most recent changes were highlighted in red.

Best regards,

Dominik Vilimek et al.

1) p. 7 "Interestingly, with increasing noise level, the efficiency of Db5 slightly decreases while in case of Db25 the efficiency increases, see Figure 4b 4c and 4d. However, SSIM metric shows that Db10 and Db25 achieved better results. In the case of higher level of decomposition similar trend could be found."

These conclusions are not supported by the figures. Differences between the curves are seen when they do not exist. For example in 4c and 4d a difference between the curves are described while in each of the figures the curves are all grouped together and are visually indistinguishable. Decrease or increase of efficiency cannot be stated with certainty these modifications are very little noticeable. These remarks hold for other figures also.

Authors' answer: Indeed, the figures were not described properly. This whole part will be corrected.

Authors' action: We rewrote following sections: Elimination of Gaussian Noise, Elimination of Salt and Pepper Noise, Elimination of Speckle Noise.

2) Depending on the criterion (correlation coefficient, MSE, PSNR, Q-index, or SSIM), the performances of the wavelets chosen are markedly different.

What is the most relevant criterion in relation to the final objective, which is the clinical interpretation of the images? this should be analyzed with the assistance of radiologists.

Authors' answer: Of the selected objective criteria, the PSNR parameter seems to be the most relevant as it is the most often used image quality metric. However, it is not recommended to use a single metric to evaluate one's results. Each of the metrics offers different point of view on the image quality and is associated with certain weaknesses and differ on their degree of sensitivity to image degradations. For example, PSNR is more sensitive to additive Gaussian noise than the SSIM as demonstrated in [1].

From representation perspective, SSIM, Q-index and correlation coefficient are easier to work with since they are normalized, whereas MSE and PSNR are not and are only showing absolute errors [2]. Moreover, SSIM was designed to take into account luminance, contrast, and structure, similarly as the human visual system [3]. This makes it theoretically the most suitable parameter to be used for this task, however, in practice, it does not have to relate to a radiologist's perception of diagnostic image quality [4]. Therefore, we used more parameters to assess the quality of the filtration and the system achieving the best results according to all (or most) of the metrics was considered as the most effective one.

It is important to mention that post-processing of ultrasound images is not a standard in current clinical practice. However, image processing can be of great benefit, especially in practices without top ultrasound devices or in cases where an increase in transmitted energy is undesirable. Moreover, current research shows that using the SSIM metric as a loss function (or component) for image enhancement using Deep Learning algorithms produces better results. Therefore, an objective evaluation based on only one parameter is unfavorable in view of future developments. Loss Function selection is a key component of today's ML and DL algorithms. Where metrics like SSIM, MSE, PSNR can be used to estimate image quality loss. Filtration is only one part of image enhancement techniques, where wavelet transformation still plays a major role [5].

Authors' action: We added the above mention to discussion section.

3) The conclusion When applied on real ultrasound images, we could observe only a slight deviation (2:2%) among different types of noise and thus this system setting can be considered as robust and effective for this application."

Should be better justified and indicate how the deviation of 2.2% is obtained

Authors' answer: Thank you for pointing this out. We have revised the conclusion and added Table 6 to discussion.

Authors' action: We modified the conclusion and demonstrated the obtained results for Daubechies family in Table 6.

4) In the abstract, the authors clam that "this study includes subjective evaluations from clinical experts, who assessed the filtration results" and "…to obtain high quality images from both the technical and clinical points of view".

These assertion on the contribution to clinical aspect are not supported by this study.

Authors' answer: Thank you for your comment. This contribution was introduced in incorrect manner and will be corrected.

Authors' action: We modified the abstract:

Wavelet transform (WT) is a commonly used method for noise suppression and feature extraction from biomedical images. The selection of WT system settings significantly affects the efficiency of denoising procedure. This comparative study analyzed the efficacy of the proposed WT system on real 292 ultrasound images from several areas of interest. The study investigates the performance of the system for different scaling functions of two basic wavelet bases, Daubechies and Symlets, and their efficiency on images artificially corrupted by three kinds of noise. To evaluate our extensive analysis, we used objective metrics, namely structural similarity index (SSIM), correlation coefficient, mean squared error (MSE), peak signal-to-noise ratio (PSNR) and universal image quality index (Q-index). Moreover, this study includes clinical insights on selected filtration outcomes provided by clinical experts. The results show that the efficiency of the filtration strongly depends on the specific wavelet system setting, type of ultrasound data, and the noise present. The findings presented may provide a useful guideline for researchers, software developers, and clinical professionals to obtain high quality images.

5) For the evaluation of clinical analysis performed by radiologists, only the case of MSK image is considered, other types of images are ignored. This is an important limitation to the interest of the paper since aiding reliable radiological analysis is the end goal of this work. Moreover, in the filtered image (c) of figure 13, we can observe that many artifacts are enhanced and can be confusing for the visual detection of veins and it is impossible to determine the right veins if we only have the filtered

image. A relevant analysis should be made by offering several radiologists to blindly analyze the images and compare their conclusions with the results obtained from the initial images.

Authors' answer: The goal of this work was to test the influence of various factors (such as wavelet system setting, or type and level of noise) on the filtration quality. This is necessary step to optimally set the system and ensure the quality of the outcomes. We discussed the evaluation aspect with radiologists. Objective evaluation in our case seems to be ideal for comparing individual settings and choosing the optimal type of mother wave, which is the aim of this article. For comparison, 292 images were created, which were artificially degraded by three variant noises of 20 levels, resulting in 17,520 images. These were subsequently filtered by systems different WT settings (16 different mother waves with two different decomposition level settings) leading to creating resulting tens of thousands of images. Based on these analyzes, we have a better idea of the robustness of each type of wave.

Due to the enormous number of images, it is beyond the scope of the article to provide subjective analysis of all of the images. This will be the goal of future work, where we aim to carry out the tests with the most suitable system setting on vast amount of data from clinical practice, where different image degradations may occur, as mentioned in the introduction. Then we aim to focus on the specific settings and impact of the clinical assessment for each image and conduct a questionnaire survey to determine the final conclusions. However, at this stage, we only wanted to provide clinical insight on selected noise and dataset. Nevertheless, we realize that we may have introduced this fact in incorrect manner thus we will made modifications in the manuscript.

Authors' action:

1) We modified the abstract:

Wavelet transform (WT) is a commonly used method for noise suppression and feature extraction from biomedical images. The selection of WT system settings significantly affects the efficiency of denoising procedure. This comparative study analyzed the efficacy of the proposed WT system on real 292 ultrasound images from several areas of interest. The study investigates the performance of the system for different scaling functions of two basic wavelet bases, Daubechies and Symlets, and their efficiency on images artificially corrupted by three kinds of noise. To evaluate our extensive analysis, we used objective metrics, namely structural similarity index (SSIM), correlation coefficient, mean squared error (MSE), peak signal-to-noise ratio (PSNR) and universal image quality index (Q-index). Moreover, this study includes clinical insights on selected filtration outcomes provided by clinical experts. The results show that the efficiency of the filtration strongly depends on the specific wavelet system setting, type of ultrasound data, and the noise present. The findings presented may provide a useful guideline for researchers, software developers, and clinical professionals to obtain high quality images.

2) We added this aspect as limitation of this study in the discussion:

The absence of extensive subjective evaluation may be considered as a limitation of this study. The blind questionnaire for image quality assessment by experts could not be conducted as the artificially degraded dataset counted a total of 17,520 images, which were subsequently filtered by various selection of mother wavelet functions leading to creating tens of thousands of images. However, using the results obtained in this study, a more robust setting can be selected and used for the evaluation tests. Moreover, the tests

should be carried out on vast amount of data from clinical practice, where different image degradations may occur, as mentioned in the introduction. Then, the questionnaire survey will be carried out to evaluate each filtrated image. However, at this stage, we only wanted to provide clinical insight on selected noise and dataset.

3) We modified the conclusion:

This study provides an extensive analysis and a quantitative evaluation of various wavelet denoising systems, their settings for different types of noise, and other effects influencing the quality of the resulting ultrasound image. The extensive analysis on in total of 17,520 images (dataset created from 292 real ultrasound images) shows that both the filtration system setting and the image content, namely type of noise and selected dataset, play a crucial role in the quality of the filtration. The performance of the tested methods was assessed by conventional objective metrics (correlation coefficient MSE, PSNR, SSIM, and Q-index). For selected filtration outcomes, we also provided clinical insights from clinical experts. The results showed that Daubechies at lower-level of decomposition achieved the best results. Namely, Db2, Db5, and Db30.

However, the obtained results also indicate that it is not possible to determine the universal type of wavelet for variant types of noise. The choice of the most effective wavelet should be tailored for a specific purpose since it depends on the type of tested ultrasound images, especially the selected area of interest, device used, and type of noise present.

[1] Hore, A., & Ziou, D. (2010, August). Image quality metrics: PSNR vs. SSIM. In *2010 20th international conference on pattern recognition* (pp. 2366-2369). IEEE.

[2] Sara, U., Akter, M., & Uddin, M. S. (2019). Image quality assessment through FSIM, SSIM, MSE and PSNR—a comparative study. *Journal of Computer and Communications*, *7*(3), 8-18.

[3] Setiadi, D. R. I. M. (2021). PSNR vs SSIM: imperceptibility quality assessment for image steganography. *Multimedia Tools and Applications*, *80*, 8423-8444.

[4] A. Mason *et al*., "Comparison of Objective Image Quality Metrics to Expert Radiologists' Scoring of Diagnostic Quality of MR Images," in *IEEE Transactions on Medical Imaging*, vol. 39, no. 4, pp. 1064-1072, April 2020, doi: 10.1109/TMI.2019.2930338

[5] Liu, JW., Zuo, FL., Guo, YX. *et al.* Research on improved wavelet convolutional wavelet neural networks. *Appl Intell* **51,** 4106–4126 (2021). https://doi.org/10.1007/s10489-020-02015-5
