# Supplementary material for: Comparative analysis of wavelet transform filtering systems for noise reduction in ultrasound images
Source: PLoS One. 2022 Jul 7;17(7):e0270745. doi: 10.1371/journal.pone.0270745 (PMC9262246; doi:10.1371/journal.pone.0270745)
Supplement: Attachment — Submitted filename: Response to Reviewers.pdf [file pone.0270745.s002.pdf]

Dear Editor,

Thank you for you feedback to our manuscript. We would like to take this opportunity and thank the reviewers for their tremendous work on reviewing our manuscript. The changes greatly improved the quality and readability of our manuscript.

Below, we are uploading point-by-point response to the comments of all reviewers. The changes of the second round of the reviews were highlighted in blue, the first round changes were kept red.

Best regards,

Dominik Vilimek et al.

Reviewer 1

Comment 1: The manuscript is now more in line with the data and results obtained. For this purpose, the authors have modified the comments of several figures to better reflect what they actually represent.

The authors also moderated their assertions concerning the radiological validity of the methods used. They recognize that the absence of extensive subjective evaluation may be considered as a limitation of this study. They argue that blind questionnaire for image quality assessment by experts could not be conducted as the artificially degraded dataset counted a total of 17,520 images, which were subsequently filtered by various selection of mother wavelet functions leading to creating tens of thousands of images. This argument is not entirely acceptable since the evaluation can be carried out on a well-chosen subset of the totality of the images. However, the limitation mentioned by the authors is sufficient in itself.

Taking into account the substantial modifications made to the manuscript, it can now be accepted for publication

Authors' answer: Thank you for all your valuable comments and time you spent reviewing our manuscript, it significantly helped in enhancing its quality. We incorporate your further comments in our future work to remove the remaining limitations.

Reviewer 2

In this paper, the authors present a comparison of wavelets transforms dedicated to denoising ultrasound images that were artificially corrupted with several types of noise. They estabish the most suitable transform depends on the type of noise but also the type of region.

The comparison is interesting and the numerous metrics and experiments could make the study very solid. However, two crucial things are missing in the paper:

Comment 1: The figures ! The 13 figures that should be present in the manuscript do not show on the PDF, as well as several parts such as section numbers. There was very probably an error of rendering. Either way this hinders a complete review as these figures are essential to understand the methodology and the results.

Authors' answer: We agree that this is confusing, however, this is the way Plos One journal's template is, please see:

https://journals.plos.org/plosone/s/file?id=wjVg/PLOSOne_formatting_sample_main_body.pdf

The figures were uploaded separately according to the guidelines and can be downloaded from the PLOS One repository (as .rar file). We are sorry about this confusion, which was not caused by us.

Authors' action: To make it easier to follow the manuscript, we are also uploading the version that has the figures incorporated in the text, this version will be uploaded as a supporting material, please see document entitled "manuscript_with_figures.pdf".

Comment 2: A clear clinical interest. The authors work on denoising images that were artificially made noisy. This noise is added mathematically, not linked to any real aspect of the acquisition. It is understandable that this allows to have as groundtruth the original image. But the original image is the one we would like to denoise. How is denoising added noise relevant to the current problems radiologists encounter in their clinical practice ? This should be made clearer.

Authors' answer: We agree that it may seem odd to use artificial noise on the images. There are different ways to test the data, as described in the Discussion. The most common approach is to use solely synthetic data or phantoms. The second is by evaluating the data visually – either by simply plotting the data before and after filtration or by using experts who would evaluate their diagnostic quality (e.g. using questionaries).

However, in our opinion, the approach used in our study is the only one suitable to test the highest amount of noise types and filter settings possible, while using the real (base) images and being able to objectively evaluate the outputs. When we would only use the real images corrupted by the noise, we would only be able to assess it subjectively – using the experts' opinions, where we would be limited by the number of images being scored and accuracy of such results (inter/intra observer reliability). We tried to make these experiments as realistic as possible: 1) by using high amount of real images acquired; 2) by using the types of noise that are most common in clinical practice (Speckle noise, Gaussian noise, and Salt

and Pepper noise); and 3) we also added a part where the clinicians had a chance to comment on the quality of the images (see section Analysis performed by radiologists (lines 315 – 360) and Fig. 13).

As a result**,** we were able to denoise the US image while preserving the diagnostically important features. However, an extensive subjective evaluation is missing in our study which we consider as a major limitation of our study, as we mentioned in discussion. The blind questionnaire for image quality assessment by experts could not be conducted due to large number of images in the dataset (17,520 subsequently filtered by various filters, i.e. tens of thousands of images to be evaluated). However, using the results obtained in this study, a more robust setting can be selected and used for the evaluation tests of a smaller dataset size.

Finally, this article only focuses on the preprocessing stage, where the aim is to reduce the significant amount of noise present in the images. In the subsequent stage, the image enhancement needs to be carried out to obtain the clinically important features and or to be able to apply the segmentation methods and so on. Therefore, the subjective evaluation by experts is crucial in these subsequent phases rather then in the pre-processing. This will be a subject of the future research.

We tried to enhance these facts in the manuscript.

Authors' action: We highlighted the above-mentioned aspects in the text as follows:

1) Introduction:

Page 2, line 25-31

There are three noise types typical for ultrasound (US) imaging: 1) Speckle noise is the most characteristic and prevalent one, it can affect important image details and may influence the intensity parameters, such as contrast; 2) Gaussian noise is caused by sensor or electronic circuit noise; 3) Salt and pepper noise occurs due to sudden changes in an image, such as memory cell failure, synchronization error during digitalization or improper function of the sensor cells.

Page 2, line 32-42

The presence of the above-mentioned noise types generally leads to degradation of visual US image quality. Thus, it is important to test the efficacy of the denoising procedure on various types of noise. In this paper we are focusing on the image preprocessing, where we often employ so-called image enhancement methods for US image noise-canceling. The preprocessing methods are aimed at noise removal and include mathematical algorithms, which can at least partially reduce the noise from US images. Image preprocessing has a substantial importance for further steps of image processing, including identification and extraction of objects of interest from ultrasound images. Images corrupted with noise or artifacts deteriorate the pixels distribution, thus decreasing performance of the image segmentation techniques such as regional and semantic segmentations.

Page 3, line 95-102

To test the highest amount of noise types and filter settings possible, we corrupted created dataset with noise generators to simulate various image impairments occurring in US images (Speckle, Gaussian, and Salt and Pepper noise). This way, we used the real US images (serving as ground truth) and were able to

objectively evaluate the outputs. In contrast to subjective evaluation by experts, which is associated with certain limitations, such as inter/extra observer disagreement.

2) Discussion:

Page 13, line 397-405

Other studies on this topic were conducted either on solely synthetic data such as [48, 55] or on a limited real dataset. For example, in [56] they used objective metrics on synthetic data and on real data, they used only 3 US images and then evaluated the performance subjectively. Subsequently, they used breast ultrasound image database containing 109 cases for experiment to demonstrate the improvement of classification by using the tested denoising algorithms. Further, the authors in [33] introduced a new wavelet type Usi and tested on 110 images from various areas, however, it was tailored for the speckle noise and thus was not tested on any other noise.

Page 15, line 450-456

However, at this stage, we only wanted to provide clinical insight on selected noise and dataset. This is because this article only focuses on the preprocessing stage, where the aim is to reduce the significant amount of noise present in the images. In the subsequent stage, the image enhancement needs to be carried out to obtain the clinically important features and or to be able to apply the segmentation methods and so on. Therefore, the subjective evaluation by experts is crucial in these subsequent phases rather than in the preprocessing. This will be a subject of the future research.
